# Cardiometabolic health of persons at risk of *APP*_V717I_ or *PSEN1*_A431E_ autosomal dominant Alzheimer’s disease in Jalisco, México

**DOI:** 10.1002/alz.088266

**Published:** 2025-01-09

**Authors:** Isaac Enrique Berumen‐Ocegueda, Adelaida Sara Minia Zepeda‐Morales, Luis Eduardo Figuera‐Villanueva, John M Ringman, Esmeralda Matute

**Affiliations:** ^1^ Instituto de Neurociencias, Universidad de Guadalajara, Guadalajara, JA Mexico; ^2^ Universidad de Guadalajara, Guadalajara, JA Mexico; ^3^ Instituto Mexicano del Seguro Social, Guadalajara, JA Mexico; ^4^ Alzheimer’s Disease Research Center, Keck School of Medicine, University of Southern California, Los Angeles, CA USA; ^5^ Departamento de Estudios en Educación, CUCSH, Universidad de Guadalajara, Guadalajara, JA Mexico; ^6^ Instituto de Neurociencias, CUCBA, Universidad de Guadalajara, Guadalajara, JA Mexico

## Abstract

**Background:**

It is known that cardiometabolic diseases are related to Alzheimer´s Disease.

**Method:**

We included 23 residents of Jalisco with at least one first‐degree relative with ADAD (ADADG). We rated the participants using the Clinical Dementia Rating (CDR) as CDR = 0 (n = 20, 87%), CDR = 0.5 (n = 2, 9%), and CDR = 2 (n = 1, 4%). Eleven participants were mutation carriers (*APP*_V717I_ = 9, *PSEN1*_A431E_ = 2). We compared the participant’s laboratory test results of the fasting plasma glucose (FPG), lipid profile, glycated hemoglobin (HbA1c), and thyroid function test (TFT) to those of 102 Jalisco residents within the same age range (CG).

**Result:**

Three (13%) carrier participants (*APP*_V717I_ = 3) reported being previously diagnosed with cardiometabolic disease (hypertension = 1, prediabetes = 1, dyslipidemia = 2). For the ADADG, the most common abnormality in the laboratory tests was dyslipidemia (n = 20, 87%; mixed dyslipidemia = 15, 66%). Other abnormalities found on laboratory tests were FPG≥126 mg/dL (n = 3, 13%), HbA1c≥6.5% (n = 7, 30%), and TFT findings compatible with hypothyroidism (n = 1, 4%). Similarly, in the CG, dyslipidemia was also the most prevalent abnormality (n = 15/22, 68%), followed by HbA1c≥6.5% (n = 6/12, 50%), and at last FPG≥126 mg/dL (n = 6/47, 13%). When comparing the ADADG to the CG, we found that the ADADG has a higher frequency of mixed dyslipidemia (χ2 = 5.02, p = .038), lower levels of free thyroxine (1.12±0.16 VS 1.28±0.19; t(37) = 2.8, p = .008), higher levels of triglycerides (187.1±75 VS 147.8±69.8; t(61) = 0.32, p = .04), and higher levels of very low‐density apolipoprotein (37.4±15 VS 28.2±11; t(43) = ‐2.3, p = .024). We did not find any difference in any measure or prevalence when comparing the mutation carrier group, the non‐carrier group, and CG.

**Conclusion:**

Dyslipidemia is the most common cardiometabolic abnormality in both ADADG and CG. The frequency of cardiometabolic abnormalities is not related to the presence of any of the two studied mutations. A follow‐up study could show if the cardiometabolic abnormalities are related to an earlier onset of ADAD.

